# Determinants of the varied profiles of *Plasmodium falciparum* infections among infants living in Kintampo, Ghana

**DOI:** 10.1186/s12936-021-03752-9

**Published:** 2021-05-29

**Authors:** Akua Kyerewaa Botwe, Felix Boakye Oppong, Stephaney Gyaase, Seth Owusu-Agyei, Muhammad Asghar, Kwaku Poku Asante, Anna Färnert, Faith Osier

**Affiliations:** 1 Kintampo Health Research Centre, Ghana Health Service, Kintampo, Ghana; 2 Division of Infectious Diseases, Department of Medicine Solna, Karolinska Institutet, Stockholm, Sweden; 3 Kenya Medical Research Institute-Wellcome Trust Research Programme, Kilifi, Kenya; 4 Institute of Health Research, University of Health and Allied Sciences, Ho, Ghana; 5 Center for Molecular Medicine, Karolinska Institutet, Stockholm, Sweden; 6 Department of Infectious Diseases, Karolinska University Hospital, Stockholm, Sweden; 7 Centre for Infectious Diseases, Parasitology, Heidelberg University Hospital, Heidelberg, Germany

**Keywords:** Infants, Malaria, *Falciparum*, Asymptomatic, Infections, Risk-of-malaria, Predictors-of-malaria, Determinants-of-malaria, Kintampo-Ghana

## Abstract

**Background:**

Understanding why some infants tolerate infections, remaining asymptomatic while others succumb to repeated symptomatic malaria is beneficial for studies of naturally acquired immunity and can guide control interventions. This study compared demographic, host and maternal factors associated with being either parasite negative or having asymptomatic infections versus developing symptomatic malaria in the first year of life.

**Methods:**

A birth cohort (n = 1264) was monitored longitudinally over two years for malaria infections in Kintampo, Ghana. Symptomatic and asymptomatic infections were detected actively through monthly home visits, complemented by passive case detection. Light microscopy was used to detect parasitaemia. Based on data from a minimum of eight monthly visits within the first year of life, infants were classified into one of four groups: “parasite negative”, “only-asymptomatic”, “only-symptomatic” or “alternating” i.e., sometimes symptomatic and other times asymptomatic. The host and maternal characteristics and demographic factors in relation to these four groups were compared.

**Results:**

The parasite negative group formed 36% of the cohort, whilst the only-symptomatic were 35%. The alternating group were 22% and the only-asymptomatic were 7% of the cohort. There were significant associations between residence, socio-economic status (SES), parity, IPTp doses, delivery place of infant and having or not having malaria parasites. Maternal factors such as early commencement and frequency of ante-natal care (ANC) were significantly higher in the parasite negative group compared to all others. ITN use in pregnancy increased the odds of infant having only asymptomatic infections (“protected against disease”). Placental malaria was more common in the groups of infants with symptomatic malaria. Urban residence was significantly higher in the parasite negative group, while birth in the malaria transmission season were significantly more common in the alternating and parasite negative groups. Risk factors for infants with symptomatic malaria included low SES, birth in private maternity homes, sickle cell normal variant, lower MUAC, reported intake of anti-malarials and increased morbidity before the first microscopic infection was detected.

**Conclusion:**

Strengthening ANC by encouraging early and regular attendance, the use of IPTp, maternal bed nets and improving the nourishment of infants help reduce the frequency of symptomatic malaria over the first year of life.

**Supplementary Information:**

The online version contains supplementary material available at 10.1186/s12936-021-03752-9.

## Background

Malaria is still a disease of public health concern, with high morbidity and mortality predominantly in children under the age of five [1]. Morbidity in children is often accompanied by signs and symptoms that range from mild to severe [2, 3]. However, infections can also be asymptomatic [4–7]. Although infants have a low risk of symptomatic malaria and frequent asymptomatic infections [5, 6], the risk factors associated with these diverse outcomes have not been studied in detail [8].

Fetal haemoglobin, maternal IgG antibodies, lactoferrin and secretory IgA in breast milk have all been reported to be associated with a lower risk of symptomatic malaria in infancy [9–12]. However, these factors are often studied separately in young infants below 6 months [8–10, 12]. Older infants from 6 to 12 months are usually studied alongside children under the age of five [7, 13, 14]. Thus, only a few studies focus on the full range of infancy, i.e., from birth up to 12 months of age, or have sufficient power to examine risk factors for symptomatic malaria [11, 15–17]. Additionally, only a handful of maternal or extrinsic factors are routinely assessed [8, 13, 15–20]. Consequently, factors that predispose infants born in malaria endemic settings to being either parasite negative or asymptomatic versus developing symptomatic malaria throughout the first year of life are not well understood.

Overcoming misclassification bias in longitudinal studies in malaria endemic areas is challenging because ascertainment of the timing and frequency of exposure (for example to infectious mosquito bites) cannot be obtained without repeated sampling; this is particularly difficult in studies involving infants and young children. Consequently, individuals classified together as “immune” to symptomatic malaria are typically either not yet exposed to mosquito bites, and thus their malaria status unclear, or asymptomatically infected, or genuinely immune i.e., clear infections without treatment [21, 22]. As a result of these potential misclassifications, the true burden of asymptomatic malaria is often underestimated, compounded further by the sensitivity of parasite detection methods [23, 24], which could overall confound the analysis of risk factors.

Ghana is currently considered by the World Health Organization (WHO) as one of eleven countries globally with the highest malaria burden [1]. Understanding the risk factors that predispose infants to remaining parasite negative or asymptomatic versus developing symptomatic malaria could complement malaria control efforts. Thus, the risk of developing malaria, diagnosed by light microscopy, among infants born to mothers in a high transmission area of Ghana was assessed, and it was observed that the incidence of all episodes of parasitaemia and clinical malaria were lowest among infants born to primigravida mothers who were negative for placental malaria [25]. Following analyses of the malaria infection profiles during the entire first year of life, a large proportion of infants were consistently parasite negative [26]. Of the infants with detectable parasitaemia, by light microscopy, a small proportion developed only asymptomatic infections which spontaneously cleared without treatment (designated “only-asymptomatic”). Significantly larger proportions developed either symptomatic malaria (designated “only-symptomatic”) or a mixture of both asymptomatic and symptomatic infections (designated “alternating”) [26]. An extension of these studies with a detailed comparison of the risk factors influencing susceptibility to symptomatic malaria using these four groups is presented here. In this study, a wide range of host, demographic and maternal factors and parasite parameters associated with being either asymptomatic or parasite negative (potentially immune) versus developing either symptomatic or alternating malaria (susceptible to disease) through the entire first year of life has been examined.

## Methods

### Study location and design

This study was carried out in the Kintampo North Municipality (KNM) and Kintampo South Districts (KSD) in the Bono East Region of Ghana. The study area covers 7162 square kilometres of land. Malaria transmission is perennial in the study area, rising from April to October [27, 28]. At the time of the study, the municipality and districts consisted of 156 villages with a resident population of approximately 1,34,970 people [29].

A birth cohort study involving pregnant women and their infants was conducted between November 2008 and January 2011 in KNM and KSD [25]. The cohort consisted of 1854 infants who were followed up in monthly scheduled home visits and unscheduled clinic visits for blood and clinical assessments [26]. The follow up ended at 12 months of age, or when the infant exited the study due to migration, death, voluntary withdrawal, or ending of the study [26].

### Sampling and data collection

At delivery, placental tissues and cord blood samples were collected in addition to recording the birth weight of the newborns as previously described [27]. Each child was visited in a monthly follow up at home by trained field staff who completed standard questionnaires documenting demographics, malaria-related parameters and previous illnesses of the ‘index’ infant. The care-givers were encouraged to bring the infant to the study clinic for health assessments, blood sampling and to complete a standard questionnaire, if the infant became unwell anytime between the home visits.

Blood samples drawn from infants during home and clinic visits were used to prepare Giemsa-stained thin and thick blood films that were examined for malaria parasites using light microscopy. Filter paper (FTA^®^) blood-spots were collected on all visits. Sickle cell variants (HbASC) and glucose-6-phosphate dehydrogenase (G6PD) deficiency amongst the first 900 infants recruited into the birth cohort study were analysed by polymerase chain reaction (PCR) and restriction fragment length polymorphisms (RFLP) based methods [28, 29]. Details of enrollment and sample collection from mothers have been previously described [30, 31].

### Longitudinal profiles of asymptomatic infections and symptomatic malaria

The longitudinal profiles of asymptomatic infections and symptomatic malaria in the first year of life have been recently reported [26]. Briefly, symptomatic malaria was adapted from the WHO and defined as a microscopy positive reading together with a history of reported fever or elevated temperature (determined as ≥ 37.5 °C) and illnesses/symptoms (such as vomiting, chills, fever, diarrhoea, cough, difficulty breathing, blood in urine or inability to suckle, drink or eat) within the past 48 h or subsequent seven days after parasite detection during either the scheduled home or unscheduled clinical visit [1, 32]. Asymptomatic infections were defined as parasite positive by microscopy at the monthly scheduled home visits with temperature < 37.5 °C and neither reported fever, illnesses nor unscheduled clinical visits in the preceding 48 h or subsequent seven days after detecting parasites [27].

From birth to twelve months of age, 1264 infants had eight or more of the monthly scheduled home visits and they were categorized into four main groups as previously described [27]. Briefly, infants who did not have malaria parasites detected throughout follow up visits were designated “parasite negative”. Infants who were asymptomatic when malaria parasites were detected on any scheduled home visits were assigned “only-asymptomatic” status. Infants who had symptomatic malaria on any occasion when parasites were detected were assigned “only-symptomatic” status, while those who had both asymptomatic infections and symptomatic malaria intermittently were assigned an “alternating” status [27].

### Statistical analyses

Chi square and Fisher’s exact tests were used to determine the association between malaria related parameters before and at first infection. Univariate and multivariate logistic regression models were used to compare risk factors of parasitemia between and within the groups of infants. Generalized estimating equations with robust standard errors were used to obtain population-averaged estimates and to address correlations present in the data [33].

To identify the determinants of anti-disease immunity in exposed infants, the only-asymptomatic group were considered potentially immune to malaria following infection, and those in the only-symptomatic and alternating groups were combined and considered susceptible to malaria. The characteristics of the potentially immune and susceptible groups were described with regards to demographic, host and maternal-related factors. The differences in the distribution of exposure variables were examined: gender, birth weight, transmission season at birth, sickle cell status, G6PD phenotypes, gestational age, anti-malarial use before the first diagnosed infection, mid-upper arm circumference (MUAC) at first infection, insecticide treated bed net (ITN) use by infant, residence, place of delivery of infant, socio-economic status (SES), ITN usage by mother during pregnancy, age of mother, placental malaria, gravidity, doses/directly observed therapy of intermittent preventive treatment during pregnancy (IPTp), number of ante-natal care (ANC) visits and number of tetanus immunizations received by mother during pregnancy.

For each group, a univariate model was first fitted to exposure variables, thereafter, significant predictors were included in multivariate analyses. Confounders included in the modelling were placental malaria, residence, ITN use by mother during pregnancy, socio-economic status (SES) and anti-malarial use before a first detected infection. SES was estimated using principal component analyses. The time to first infection was assessed per group using Kaplan–Meier curves, and to identify the effects of haemoglobinopathies. The second child of every twin birth was excluded to prevent clustering effects. Estimates with p-values < 0.05 were considered statistically significant. Data were analysed using Stata Version 14 (STATA Corporation, College Station, TX).

## Results

### Characteristics of the cohort

**Fig. 1:**
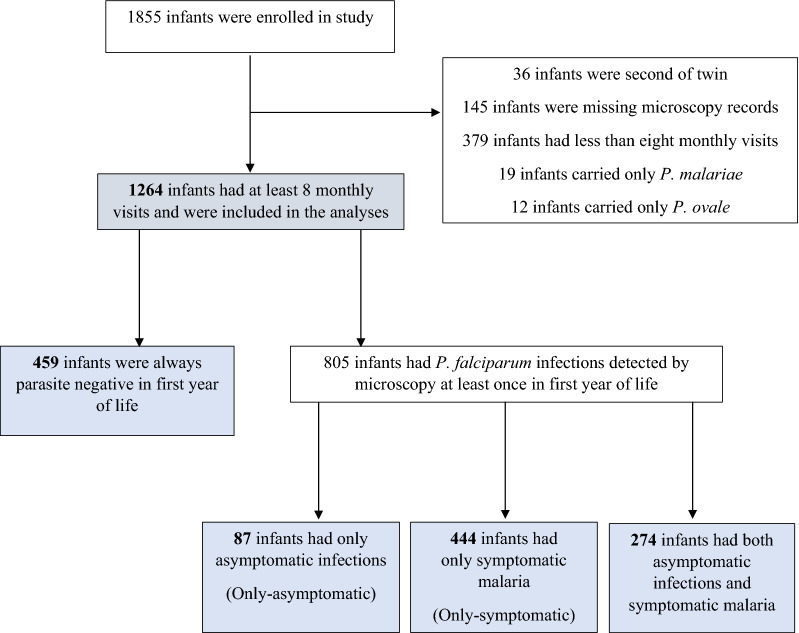
Groups of infants studied. The flow chart shows how the groups of infants examined in this study were obtained from the birth cohort

The dynamics of infections, and parasite densities have been previously described in more detail for the cohort [26]. Briefly, among the 1264 infants, 459 (36%) were repeatedly “parasite negative”, 87 (7%) were “only-asymptomatic”, 444 (35%) were “only-symptomatic” and 274 (22%) had both asymptomatic and symptomatic infections intermittently and referred to as the “alternating” group (Fig. 1). Infants in the alternating group had a higher total number and cumulative incidence of infection and were younger at their first infection compared to the other groups of infants having malaria parasites. The parasite densities were lowest for the only-asymptomatic group and highest for the only-symptomatic group [26].

### Differences in host, maternal and parasite parameters between the four groups of infants

Examination of infants before their first infection showed that among those having malaria parasites, the only-asymptomatic group had fewer reports of past fevers, less elevated temperature and illnesses, compared to the only-symptomatic or alternating groups (Table 1).

**Table 1:** Malaria related parameters before and at first infection

Characteristic	Level	Only-asymptomatic n = 87 n (%)	Only-symptomatic n = 444 n (%)	Alternating n = 274 n (%)	Overall p-value	Only-asymptomatic vs only-symptomatic p-value	Only-asymptomatic vs alternating p-value
Clinical visits before the first infection	Yes	86 (98.9)	444 (100)	271 (98.9)	**0.043**	0.164	0.670
	No	1 (1.1)	0 (0.0)	3 (1.1)			
Reported fever^a^ before the first infection	Yes^a^	38 (43.7)	281 (63.3)	118 (43.1)	**< 0.001**	**0.001**	0.920
	No	49 (56.3)	163 (36.7)	156 (56.9)			
Elevated^b^ temperature before the first infection	Yes^b^	12 (13.8)	117 (26.4)	39 (14.2)	**< 0.001**	**0.013**	0.918
	No	75 (86.2)	327 (73.6)	235 (85.8)			
Illness before first infection	Yes	34 (39.1)	308 (69.4)	126 (46.0)	**< 0.001**	**< 0.001**	0.259
	No	53 (60.9)	136 (30.6)	148 (54.0)			
Anti-malarial use before the first infection	Yes	3 (3.4)	279 (62.8)	64 (23.4)	**< 0.001**	**< 0.001**	**< 0.001**
	No	84 (96.6)	165 (37.2)	210 (76.6)			
MUAC (cm) at first infection	Median (IQR)	14.2 (13.2, 15.3)	14.3 (13.5, 15.0)	14.0 (13.2, 15.0)	**0.040**	0.849	0.217

^a^Fever is reported in past 48 h by mother on clinical visits

^b^Elevated temperature is measured temperature above 37.5 °C on the day of home or clinical visit

MUAC = mid-upper arm circumference (measure for assessing malnutrition)

There were significant differences in past fevers, elevated temperature and illnesses when the only-asymptomatic and only-symptomatic were compared, but not when the only-asymptomatic and alternating infants were compared (Table 1). Caregivers reported lower intake of anti-malarials before the first diagnosed infection for infants in the only-asymptomatic group (3%) compared to the alternating (23%) or only-symptomatic group (63%) (p < 0.001) (Table 1). The parasite negative group had the highest residency in urban areas (28%) (p < 0.001) while among infants with malaria parasites the only-asymptomatic group had the highest residency in urban areas (p < 0.001) (Additional file 1). The distribution of other factors including SES, ANC visits, placental malaria, place of birth, frequency of tetanus immunizations and IPTp doses were significantly dis-similar between the parasite negative versus any other infant group (Additional files 1, 2).

### Immune (only-asymptomatic) versus susceptible (alternating and only-symptomatic)

Fevers and illnesses before the first detected infection were significantly lower in the only-asymptomatic group compared to the susceptible group. Anti-malarial use before the first detected infection was also lower in the immune group (3%) compared to the susceptible group (47.8%) (p < 0.001) (Table 2). There was a significant association between SES, placental malaria, ITN usage during pregnancy or IPTp usage and having only-asymptomatic infections or being susceptible to malaria (Table 2). While few infants in the only-asymptomatic group were in poor, poorer and most poor (low SES) households, more infants in the susceptible group were in the low SES households (p < 0.001) (Table 2). The proportion of mothers who did not have placental malaria within the only-asymptomatic group was higher (67%) than within the susceptible group (61%) and chronic placental malaria was absent in the only-asymptomatic group (p = 0.010) (Table 2).

**Table 2:** Distribution of significant factors in immune versus susceptible infants

Characteristic	Level	Only-asymptomatic n = 87 n (%)	Only-symptomatic and alternating n = 718 n (%)	p-value
Reported fever before first infection	Yes	38 (43.7)	399 (55.6)	**0.035**
	No	49 (56.3)	319 (44.4)	
Illness before first infection	Yes	34 (39.1)	434 (60.4)	**< 0.001**
	No	53 (60.9)	284 (39.6)	
Anti-malarial use before first infection	Yes	3 (3.4)	343 (47.8)	**< 0.001**
	No	84 (96.6)	375 (52.2)	
Socio-economic status	Least poor	20 (23.0)	68 (9.5)	**< 0.001**
	Less poor	19 (21.8)	124 (17.3)	
	Poor	20 (23.0)	160 (22.3)	
	Poorer	17 (19.5)	167 (23.3)	
	Most poor	11 (12.6)	199 (27.7)	
IPTp, DOTs	Yes	73 (90.1)	660 (95.8)	**0.024**
	No	8 (9.9)	29 (4.2)	
Overall ITN use during pregnancy	Yes	52 (61.2)	345 (48.6)	**0.028**
	No	33 (38.8)	365 (51.4)	
ITN use during pregnancy in rural areas	Yes	43 (59.7)	299 (46.6)	**0.035**
	No	29 (40.3)	342 (53.4)	
Placental malaria overall	Negative	58 (66.7)	436 (60.7)	**0.010**
	Past	23 (26.4)	227 (31.6)	
	Chronic	0 (0.0)	38 (5.3)	
	Acute	6 (6.9)	17 (2.4)	
Placental malaria in rural areas	Negative	51 (68.9)	398 (61.4)	**0.021**
	Past	18 (24.3)	199 (30.7)	
	Chronic	0 (0.0)	35 (5.4)	
	Acute	5 (6.8)	16 (2.5)	

The bold emphasis of p-values indicate significant differences in exposure variables in relation to the outcomes which were compared

IPTp = intermittent preventive treatment during pregnancy; DOTs = directly observed therapy; ITN = insecticide treated bed net

**Fig. 2:**
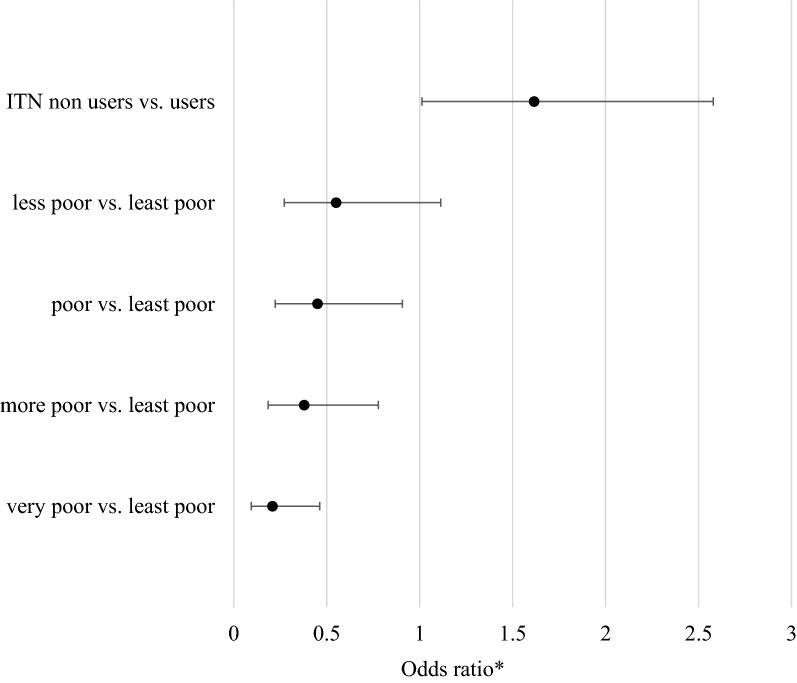
Factors significantly affecting the risk of immune compared to being susceptible to malaria. Increased probability of infants having only asymptomatic infections (immune) if mother used ITN during pregnancy relative to ITN non-use (as reference = 1); and decreasing probability of being immune to disease as socioeconomic status (SES) decreases, with least poor as reference. *Adjusted for ITN use, socioeconomic status, residence and placental malaria

ITN use during pregnancy was higher among mothers of the only-asymptomatic group (61.2%) compared to usage by mothers of the susceptible group (48.9%) (p = 0.028); and fewer mothers of the only-asymptomatic group received IPTp DOTs (90%) compared to the susceptible group (96%) (p = 0.024) (Table 2). After adjusting for placental malaria, SES, ITN usage during pregnancy and residence, and compared to infants from least poor (highest SES) households, those from most poor (lowest SES) households had reduced odds (adjusted OR 0.21, 95% CI 0.09–0.46; p < 0.001) of having only-asymptomatic infections (Fig. 2). Compared to infants whose mothers did not use ITN during pregnancy, those whose mothers used ITN had an increased odds (adjusted OR 1.62, 95% CI 1.01–2.58; p = 0.044) of having only-asymptomatic infections (‘immune to disease’) (Fig. 2).

### Parasite negative versus parasite positive

The only-asymptomatic, only-symptomatic and alternating groups were combined as a single group of infants with parasite infections and compared with the parasite negative group. Significant associations between residence, SES, parity, IPTp doses or the place of delivery of infant and having or not having parasites were observed (Additional file 3). Fewer infants within the parasite negative group (10%) were resident in rural areas compared to the positive group (28%) (p < 0.001) (Additional file 3). While few infants in the parasite negative group had low SES, more infants in the parasite positive group had low SES (p < 0.001). The proportion of primigravid within the parasite negative was more (19%) compared to the primigravid in the parasite positive group (17%) (p = 0.024). More pregnant women received three IPTp doses in the parasite negative group (61%) compared to the parasite positive group (58%) (p = 0.004), and more infants of the parasite negative group (70%) compared to the parasite positive group (58%) were delivered in a health facility (p = 0.001) (Additional file 3).

### Risk of having a specific profile of infection

Compared to the only-asymptomatic group, the risk of having an infection was lower for the only-symptomatic group (adjusted OR 0.77, 95% CI 0.62–0.96; p = 0.019) than the alternating group (adjusted OR 2.21, 95% CI 1.80–2.72; p < 0.001) (Table 3).

**Table 3:** Relative risk of only-symptomatic or alternating pattern in the first year of life

Malaria profile	Crude OR (95% CI)	p-value	*Adjusted OR (95% CI)	p-value
Only-asymptomatic	1		1	
Only-symptomatic	0.79 (0.64–0.96)	0.019	0.77 (0.62–0.96)	0.019
Alternating	2.33 (1.89–2.86)	< 0.001	2.21 (1.80–2.72)	< 0.001

^*^ = Adjusted for residence, ITN use, anti-malarial use before a first infection, socioeconomic status and placental malaria

In the only-asymptomatic group, infants from low SES households had an increased risk of infection compared to those from the highest SES households (Table 4). In the only-symptomatic group, infants with higher MUAC had a reduced risk of symptomatic malaria (adjusted OR 0.98, 95% CI 0.97–0.99; p = 0.005), while infants from low SES households compared to those from the highest SES households had increased risk of infection (Table 4). The factors which reduced the risk of alternating between asymptomatic infections and symptomatic malaria were urban residence compared to rural (adjusted OR 0.76, 95% CI 0.60–0.96; p = 0.022) and higher MUAC (adjusted OR 0.98, 95% CI 0.97–0.99; p < 0.001). The age of mother somewhat increased the risk of infection for infants in the alternating group (adjusted OR 1.01, 95% CI 1.00–1.03; p = 0.047) (Table 4). Similar to the other groups of infants having malaria parasites, low socio-economic status compared to the highest increased the risk of infections for infants in the alternating group (Table 4).

**Table 4:** Risk of infection within the group of infants

Variable	Category	Only-asymptomatic infection				Only-symptomatic infection				Alternating			
		OR (95% CI)	p-value	*AdjustedOR (95%CI)	p-value	OR (95% CI)	p-value	*AdjustedOR (95% CI)	p-value	OR (95% CI)	p-value	*AdjustedOR (95%CI)	p-value
Residence	Rural	1		1		1		1		1		1	
	Urban	0.59 (0.42–0.82)	**0.001**	0.81 (0.59–1.11)	0.193	0.92 (0.74–1.16)	0.499	0.95 (0.72–1.26)	0.731	0.77 (0.63–0.94)	**0.011**	0.76 (0.60–0.96)	**0.022**
ITN^a^ usage by mother during pregnancy	Yes	1		1		1		1		1		1	
	No	0.91 (0.63–1.34)	0.645	0.91 (0.62–1.33)	0.610	1.04 (0.91–1.19)	0.540	1.00 (0.87–1.13)	0.927	0.96 (0.82–1.12)	0.612	0.93 (0.79–1.09)	0.361
Placental malaria	Negative	1		1		1		1		1		1	
	Positive	1.06 (0.70–1.61)	0.789	1.01 (0.65–1.56)	0.969	1.04 (0.91–1.19)	0.540	1.03 (0.90–1.17)	0.698	0.97 (0.83–1.14)	0.724	1.06 (0.89–1.25)	0.510
SES^b^	Least poor	1		1		1		1		1		1	
	Less poor	1.05 (0.74–1.49)	0.797	1.12 (0.78–1.61)	0.527	1.03 (0.81–1.32)	0.795	0.97 (0.75–1.24)	0.784	1.16 (0.88–1.52)	0.298	1.19 (0.91–1.56)	0.210
	Poor	1.83 (1.12–2.99)	**0.015**	1.83 (1.09–3.05)	**0.022**	1.37 (1.07–1.75)	**0.011**	1.32 (1.03–1.70)	**0.029**	1.36 (1.03–1.80)	**0.028**	1.31 (1.01–1.71)	**0.044**
	Poorer	1.64 (1.08–2.47)	**0.019**	1.64 (1.05–2.54)	**0.029**	1.32 (1.02–1.69)	**0.032**	1.22 (0.93–1.59)	0.152	1.31 (1.00–1.73)	**0.052**	1.31 (1.00–1.72)	**0.052**
	Most poor	2.96 (1.64–5.37)	**< 0.001**	2.98 (1.67–5.34)	**< 0.001**	1.52 (1.19–1.94)	**0.001**	1.42 (1.09–1.85)	**0.009**	1.47 (1.13–1.91)	**0.004**	1.41 (1.08–1.84)	**0.011**
Anti-malarial use before infection	No	1		1		1		1		1		1	
	Yes	0.82 (0.54–1.24)	0.347	0.93 (0.64–1.36)	0.701	0.92 (0.80–1.05)	0.217	1.00 (0.87–1.16)	0.992	1.05 (0.88–1.25)	0.591	1.13 (0.94–1.35)	0.193
MUAC^c^		0.99 (0.98–1.01)	0.273	–	–	0.98 (0.97–0.99)	**0.004**	0.98 (0.97–0.99)	**0.005**	0.98 (0.97–0.99)	**< 0.001**	0.98 (0.97–0.99)	**< 0.001**
Age of mother		1.03 (1.00–1.06)	0.062	–	–	1.00 (1.00–1.01)	0.363	–	–	1.01 (1.00–1.02)	**0.046**	1.01 (1.00–1.03)	**0.047**
ANC^d^ visits		0.98 (0.87–1.11)	0.735	–	–	0.96 (0.93–1.00)	**0.030**	0.98 (0.96–1.00)	0.103	0.97 (0.93–1.01)	0.155	–	–

The bold emphasis of p-values indicate significant differences in exposure variables in relation to the outcomes which were compared

*ITN*   insecticide treated bed-net; *SES*  socio-economic status; *MUAC*  mid-upper arm circumference; *ANC* antenatal care

*Adjusted for residence, ITN use, antimalarial usage before first infection, SES, placental malaria, and gestation

### Association between infections and haemoglobinopathies

Among infants in all three infection groups namely: only-asymptomatic, only-symptomatic and the alternating, the survival curves for infants with normal red blood cells, compared to those with sickle cell trait or disease were similar prior to three months of age (Additional file 4a–c). In the alternating group, the risk of first malaria infection was significantly higher among infants with normal red blood cells compared to those with sickle cell trait or disease (HR: 1.52, 95% CI 1.04–2.21, p value = 0.029) (Additional file 4a). The risk of first malaria infection were similar when comparing infants with G6PD deficiency to infants with the normal enzyme in all the groups of infants with parasites (Additional file 5a–c).

## Discussion

A longitudinally monitored birth cohort was used for detailed analyses of the host, demographic and maternal factors associated with having only asymptomatic infections or being parasite negative versus having symptomatic malaria, throughout the first year of life in a high malaria transmission setting in Ghana. Compared to having “only-asymptomatic” status, infants were twice more likely to have an “alternating” status and somewhat less likely to have “only-symptomatic” status through the first year of life.

Regular ANC attendance, where education on safe pregnancy practices is also provided, may have improved mothers’ personal malaria prevention strategies, as mothers of the infants who were parasite negative attended ANC more frequently and ANC attendance was protective against symptomatic malaria. A similar observation has been made in Uganda, East Africa, where maternal sensitization on malaria prevention was associated with a reduced odds of infection in infants [34]. Additionally, in this study, mothers of the infants who were parasite negative started ANC earlier and received more doses of tetanus immunizations compared to the other groups of infants. Further, ITN use during pregnancy was associated with infant being parasite negative or having only asymptomatic infections in the first year of life.

Placental malaria was not a risk of being in any of the parasite positive groups. Nevertheless, while the infants within the only-symptomatic group had the highest proportion of mothers with a past placental malaria, those in the alternating group had the highest proportion of mothers with chronic placental malaria; and the parasite negative and only-asymptomatic groups had the highest proportion of mothers without placental malaria and lowest proportion of mothers with chronic placental malaria. Some studies have showed that the infants born to mothers with placental malaria are less protected against malaria [16, 35], or that compared to infants with passively transferred maternal IgG, those without have a reduce risk of symptomatic malaria [18, 19, 36], but others did not show such associations [5, 8, 15]. Studies focused on maternal antibodies and malaria susceptibility during infancy may achieve consistency by stratifying according to how infections are presented longitudinally among infants.

The delivery place of an infant may have important implications for malaria control, as most infants born in private maternity homes belonged to the susceptible groups and majority of those born in a public/state-owned health facility were in the parasite negative and only-asymptomatic groups. Further, infants in the parasite negative group had a high SES and a mother aged above 20 years who received three doses of SP IPTp and with a maximum of two other children. Older mothers with a higher SES, fewer children and more compliant with IPTp are probably better able to care for themselves and their children. Similar to these findings, maternal wealth has been shown to reduce parasitaemia [34, 37]. On the contrary, no significant association between IPTp use and risk of malaria during infancy has been shown [38]. Multiple longitudinal studies in different malaria transmission settings which demonstrate that the combined interactions between IPTp usage, delivery place, maternal age, parity and placental malaria have effects during infancy may be relevant to malaria control strategies.

Host characteristics were similar between the parasite negative and only-asymptomatic groups, as well as between the alternating and only-symptomatic groups, and thus the latter groups (alternating and only-symptomatic) were considered as a single susceptible group and compared to the only-asymptomatic group in some analyses. This decision was also based on the fact that parasites were detected only by light microscopy. Given the limited sensitivity of light microscopy compared to polymerase chain reaction (PCR) based detection [39], it was expected that some of the infants classified as parasite negative may harbour parasites below the detection limit of microscopy. Thus, the analyses highlighting the differences between the only-asymptomatic and the groups susceptible to symptomatic malaria are considered optimal based on the current WHO gold standard (microscopy) of malaria diagnoses globally [1].

Within the groups of infants having parasites, low SES increased risk of infection, while increasing MUAC (a marker of nourishment) and urban residence reduced the risk. Further, among the only-symptomatic group, higher morbidity (more reported fevers, elevated temperature and illnesses) and more reported (by caregivers) intake of anti-malarials by infant before the first detectable infection was observed. Additionally, the only-symptomatic group had higher residence in rural areas compared to the parasite negative or only-asymptomatic groups. These findings support other reports which show that symptomatic malaria in infants tend to flourish where there is poverty and interventions which focus on improving SES or reducing infant morbidities could positively impact control efforts [34, 40]. Nonetheless, the frequency of morbidities before the first detectable microscopic infection was significantly low and may be an indicator of being less susceptible to symptomatic malaria during the first months of life.

In the groups of infants having parasites, the protective effects of sickle cell trait were observed beginning from three months of age, where maternal antibodies begin to wane [41], thus leading to increased infections [4]. Infants having the alternating sequence pattern of infection and sickle cell trait or disease had their first infections latter than their counterparts with normal red blood cells, thus supporting the observation that the presence of sickle cell trait protects against or delays the onset of symptomatic malaria [42–44]. The results also showed that the time to a first infection was similar between infants with G6PD deficiency trait and those with the normal enzyme in all groups. A protective association between G6PD deficiency and malaria through the first year of life may be easier to detect in studies which focus on sex-linkage or malaria-severity [45].

Regarding demographic factors, Apinjoh et al*.* [17] showed that infants born in the dry (low) malaria transmission season are protected while those born in the wet (high) malaria transmission season are more susceptible to malaria. Similarly, in this study infants who were in only-asymptomatic group (immune protection against disease) were mostly born in the low transmission season while those with the alternating sequence pattern of infection were mostly born in the high malaria transmission season. Nevertheless, when immunity (protection against parasites) is perceived as being parasite negative, an opposite observation with births occurring more frequently during the high (versus low) malaria transmission season in the parasite negative group is made. The infants in the parasite negative group were more likely to live in urban areas and had the highest socio-economic status (SES), suggesting that despite birth in a high malaria transmission season, exposure to mosquitoes may be less and consequently malaria parasites may be absent, if the infant is resident in an urban area and lives in a high SES household.

By restricting the analyses to those with at least eight samples, a selection bias towards mothers/infants that better complied to the monthly follow ups, which could have influenced the selection and number of infants examined per group in this study was contemplated. Nevertheless, the sample size analysed (1264 infants) provided sufficient statistical power to detect an effect and, the interpretations in this study are relevant in the context of the WHO’s gold standard (light microscopy) for the diagnosis of malaria globally [1]. In addition to the impracticability of examining by molecular methods all the monthly parasite positive and negative samples from 1264 infants, another potentially important limitation is the lack of data on the HIV status of infants involved in this longitudinal cohort analyses. However, this limitation may not have significantly affected the study outcomes because the HIV incidence of all ages in 2010 was low (1.3 new cases/1000 individuals; CI 1.1–1.6) while 1300 (CI 1000–1700) new HIV infections were averted due to preventive mother to child transmission strategies in Ghana [46]. Although the analyses of samples were not made using molecular methods, the strength of this study lies in the power of cohort, the detailed analyses of multiple host, demographic and maternal factors, the frequency of scheduled sampling and the duration of monitoring over the entire first year of life.

## Conclusions

Of all the potential risk factors assessed, poor socio-economic status and maternal ANC attendance, ITN and IPTp use were most consistently associated with symptomatic malaria. Importantly, although raising socio-economic status is arguably a longer-term endeavour, simple measures such as encouraging maternal ANC attendance, ITN and IPTp use have significantly positive impact on infant outcomes not only at birth, but throughout the first year of life. This study supports the continued implementation of these malaria control measures.

## Supplementary Information

**Additional file 1: Table S1.** Distribution of host characteristics between groups of infants.

**Additional file 2: Table S2.** Distribution of maternal and pregnancy factors between groups of infants.

**Additional file 3: Table S3.** Parasite negative versus only-asymptomatic*, only-symptomatic* and alternating*.

**Additional file 4: Figure S1.** Sickle cell variation and time to first malaria infection.

**Additional file 5: Figure S2.** G6PD deficiency and time to first malaria infection.

## Data Availability

The datasets used and/or analysed during the current study are available from the corresponding author on reasonable request.

## Abbreviations

ANC: Antenatal care
G6PD: Glucose-6-phosphate dehydrogenase
IPTp: Intermittent preventive treatment during pregnancy
ITN: Insecticide treated bed net
MUAC: Mid-upper arm circumference
PCR: Polymerase chain reaction
SES: Socio-economic status

## References

[1] WHO. World Malaria Report. Geneva, World Health Organization; 2019. https://www.who.int/publications-detail/world-malaria-report-2019. Accessed 22 Jan 2020.

[2] Idro R, Bitarakwate E, Tumwesigire S, John CC (2005). Clinical manifestations of severe malaria in the highlands of southwestern Uganda. Am J Trop Med Hyg.

[3] Marsh K, Forster D, Waruiru C, Mwangi I, Winstanley M, Marsh V (1995). Indicators of life-threatening malaria in African children. N Engl J Med.

[4] Natama HM, Rovira-Vallbona E, Some MA, Zango SH, Sorgho H, Guetens P (2018). Malaria incidence and prevalence during the first year of life in Nanoro, Burkina Faso: a birth-cohort study. Malar J.

[5] Wagner G, Koram K, McGuinness D, Bennett S, Nkrumah F, Riley E (1998). High incidence of asymptomatic malara infections in a birth cohort of children less than 1 year of age in Ghana, detected by multicopy gene polymerase chain reaction. Am J Trop Med Hyg.

[6] Franks S, Koram KA, Wagner GE, Tetteh K, McGuinness D, Wheeler JG (2001). Frequent and persistent, asymptomatic *Plasmodium falciparum* infections in African infants, characterized by multilocus genotyping. J Infect Dis.

[7] Ceesay SJ, Koivogui L, Nahum A, Taal MA, Okebe J, Affara M (2015). Malaria prevalence among young infants in different transmission settings. Africa Emerg Infect Dis.

[8] Riley EM, Wagner GE, Ofori MF, Wheeler JG, Akanmori BD, Tetteh K (2000). Lack of association between maternal antibody and protection of African infants from malaria infection. Infect Immun.

[9] Amaratunga C, Lopera-Mesa TM, Brittain NJ, Cholera R, Arie T, Fujioka H (2011). A role for fetal hemoglobin and maternal immune IgG in infant resistance to *Plasmodium falciparum* malaria. PLoS ONE.

[10] Pasvol G, Weatherall DJ, Wilson RJ (1977). Effects of foetal haemoglobin on susceptibility of red cells to *Plasmodium falciparum*. Nature.

[11] Achidi EA, Perlmann H, Salimonu LS, Perlmann P, Walker O, Asuzu MC (1995). A longitudinal study of seroreactivities to *Plasmodium falciparum* antigens in Nigerian infants during their first year of life. Acta Trop.

[12] Kassim OO, Ako-Anai KA, Torimiro SE, Hollowell GP, Okoye VC, Martin SK (2000). Inhibitory factors in breastmilk, maternal and infant sera against in vitro growth of *Plasmodium falciparum* malaria parasite. J Trop Pediatr.

[13] Coalson JE, Walldorf JA, Cohee LM, Ismail MD, Mathanga D, Cordy RJ (2016). High prevalence of *Plasmodium falciparum* gametocyte infections in school-age children using molecular detection: patterns and predictors of risk from a cross-sectional study in southern Malawi. Malar J.

[14] Njama-Meya D, Kamya MR, Dorsey G (2004). Asymptomatic parasitaemia as a risk factor for symptomatic malaria in a cohort of Ugandan children. Trop Med Int Health.

[15] Hogh B, Marbiah NT, Burghaus PA, Andersen PK (1995). Relationship between maternally derived anti-*Plasmodium falciparum* antibodies and risk of infection and disease in infants living in an area of Liberia, west Africa, in which malaria is highly endemic. Infect Immun.

[16] Bonner PC, Zhou Z, Mirel LB, Ayisi JG, Shi YP, van Eijk AM (2005). Placental malaria diminishes development of antibody responses to *Plasmodium falciparum* epitopes in infants residing in an area of western Kenya where *P. falciparum* is endemic. Clin Diagn Lab Immunol.

[17] Apinjoh TO, Anchang-Kimbi JK, Mugri RN, Njua-Yafi C, Tata RB, Chi HF (2015). Determinants of infant susceptibility to malaria during the first year of life in south western Cameroon. Open Forum Infect Dis.

[18] Sehgal VM, Siddjiqui WA, Alpers MP (1989). A seroepidemiological study to evaluate the role of passive maternal immunity to malaria in infants. Trans R Soc Trop Med Hyg.

[19] Kangoye DT, Nebie I, Yaro JB, Debe S, Traore S, Ouedraogo O (2014). *Plasmodium falciparum* malaria in children aged 0–2 years: the role of foetal haemoglobin and maternal antibodies to two asexual malaria vaccine candidates (MSP3 and GLURP). PLoS ONE.

[20] Muirhead-Thomson RC (1951). The distribution of anopheline mosquito bites among different age groups; a new factor in malaria epidemiology. BMJ.

[21] Marsh K, Kinyanjui S (2006). Immune effector mechanisms in malaria. Parasite Immunol.

[22] Proietti C, Verra F, Bretscher MT, Stone W, Kanoi BN, Balikagala B (2013). Influence of infection on malaria-specific antibody dynamics in a cohort exposed to intense malaria transmission in northern Uganda. Parasite Immunol.

[23] Brown AE, Kain KC, Pipithkul J, Webster HK (1992). Demonstration by the polymerase chain reaction of mixed *Plasmodium falciparum* and *P. vivax* infections undetected by conventional microscopy. Trans R Soc Trop Med Hyg.

[24] Goncalves BP, Drakeley C, Bousema T (2016). Infectivity of microscopic and submicroscopic malaria parasite infections in areas of low malaria endemicity. J Infect Dis.

[25] Asante KP, Owusu-Agyei S, Cairns M, Dodoo D, Boamah EA, Gyasi R (2013). Placental malaria and the risk of malaria in infants in a high malaria transmission area in Ghana: a prospective cohort study. J Infect Dis.

[26] Botwe AK, Owusu-Agyei S, Asghar M, Hammar U, Oppong FB, Gyaase S (2020). Profiles of *Plasmodium falciparum* infections detected by microscopy through the first year of life in Kintampo a high transmission area of Ghana. PLoS ONE.

[27] Owusu-Agyei S, Nettey OE, Zandoh C, Sulemana A, Adda R, Amenga-Etego S (2012). Demographic patterns and trends in central Ghana: baseline indicators from the Kintampo health and demographic surveillance system. Glob Health Action.

[28] Dery DB, Brown C, Asante KP, Adams M, Dosoo D, Amenga-Etego S (2010). Patterns and seasonality of malaria transmission in the forest-savannah transitional zones of Ghana. Malar J.

[29] Carter N, Pamba A, Duparc S, Waitumbi JN (2011). Frequency of glucose-6-phosphate dehydrogenase deficiency in malaria patients from six African countries enrolled in two randomized anti-malarial clinical trials. Malar J.

[30] Waterfall CM, Cobb BD (2001). Single tube genotyping of sickle cell anaemia using PCR-based SNP analysis. Nucleic Acids Res.

[31] Asante KP, Owusu-Agyei S, Cairns M, Boamah E, Manu G, Twumasi M (2016). Non-malaria fevers in a high malaria endemic area of Ghana. BMC Infect D.

[32] WHO. Malaria Terminology. Geneva, World Health Organization; 2018. http://www.who.int/malaria/visual-refresh/en/WHO/HTM/GMP/2016.6. Accessed on 24 Sept 2019.

[33] Liang K-Y, Zeger SL (1986). Longitudinal data analysis using generalized linear models. Biometrika.

[34] Wanzira H, Katamba H, Okullo AE, Agaba B, Kasule M, Rubahika D (2017). Factors associated with malaria parasitaemia among children under 5 years in Uganda: a secondary data analysis of the 2014 malaria indicator survey dataset. Malar J.

[35] Le Port A, Watier L, Cottrell G, Ouedraogo S, Dechavanne C, Pierrat C (2011). Infections in infants during the first 12 months of life: role of placental malaria and environmental factors. PLoS ONE.

[36] Khattab A, Chia YS, May J, Le Hesran JY, Deloron P, Klinkert MQ (2007). The impact of IgG antibodies to recombinant *Plasmodium falciparum* 732var CIDR-1alpha domain in mothers and their newborn babies. Parasitol Res.

[37] Okiring J, Olwoch P, Kakuru A, Okou J, Ochokoru H, Ochieng TA (2019). Household and maternal risk factors for malaria in pregnancy in a highly endemic area of Uganda: a prospective cohort study. Malar J.

[38] Kakuru A, Staedke SG, Dorsey G, Rogerson S, Chandramohan D (2019). Impact of *Plasmodium falciparum* malaria and intermittent preventive treatment of malaria in pregnancy on the risk of malaria in infants: a systematic review. Malar J.

[39] Morassin B, Fabre R, Berry A, Magnaval JF (2002). One year’s experience with the polymerase chain reaction as a routine method for the diagnosis of imported malaria. Am J Trop Med Hyg.

[40] Mathanga DP, Tembo AK, Mzilahowa T, Bauleni A, Mtimaukenena K, Taylor TE (2016). Patterns and determinants of malaria risk in urban and peri-urban areas of Blantyre, Malawi. Malar J.

[41] Murungi LM, Sonden K, Odera D, Oduor LB, Guleid F, Nkumama IN (2017). Cord blood IgG and the risk of severe *Plasmodium falciparum* malaria in the first year of life. Int J Parasitol.

[42] Crompton PD, Traore B, Kayentao K, Doumbo S, Ongoiba A, Diakite SA (2008). Sickle cell trait is associated with a delayed onset of malaria: implications for time-to-event analysis in clinical studies of malaria. J Infect Dis.

[43] Williams TN, Mwangi TW, Wambua S, Alexander ND, Kortok M, Snow RW (2005). Sickle cell trait and the risk of *Plasmodium falciparum* malaria and other childhood diseases. J Infect Dis.

[44] Goncalves BP, Huang CY, Morrison R, Holte S, Kabyemela E, Prevots DR (2014). Parasite burden and severity of malaria in Tanzanian children. N Engl J Med.

[45] Guindo A, Fairhurst RM, Doumbo OK, Wellems TE, Diallo DA (2007). X-linked G6PD deficiency protects hemizygous males but not heterozygous females against severe malaria. PLoS Med.

[46] Ghana UNAIDS Country Fact-sheet. 2018. https://www.unaids.org/en/regionscountries/countries/ghana. Accessed 15 March 2019.

