# Supplementary material for: Determinants of the varied profiles of Plasmodium falciparum infections among infants living in Kintampo, Ghana
Source: Malar J. 2021 May 29;20:240. doi: 10.1186/s12936-021-03752-9 (PMC8164218; doi:10.1186/s12936-021-03752-9)
Supplement: Supplementary file 1 — Additional file 1: Table S1. Distribution of host characteristics between groups of infants. [file 12936_2021_3752_MOESM1_ESM.pdf]

Additional file 1. Distribution of host characteristics between groups of infants

Characteristic	Level	Parasite negative N = 459 n (%)	Only-asymptomatic N = 87 n (%)	Only-symptomatic N = 444 n (%)	Alternating N = 274 n (%)	Overall P-value	Parasite negative vs. Only-symptomatic P- value	Parasite negative vs. Only-asymptomatic P- value	Parasite negative vs. Alternating P-value	Only-asymptomatic vs. Only-symptomatic P- value	Only-asymptomatic vs. Alternating P- value
**Sex**	Male	242 (52.7)	40 (46.0)	246 (55.4)	128 (46.7)	0.090	0.419	0.248	0.115	0.107	0.904
	Female	217 (47.3)	47 (54.0)	198 (44.6)	146 (53.3)						
**Birth weight (kg)**	Median (IQR [b])	3.00 (2.70, 3.40)	3.00 (2.60, 3.40)	3.00 (2.70, 3.30)	3.00 (2.70, 3.30)	0.120	0.255	0.401	**0.018**	0.870	0.540
	≥ 2.5 kg	425 (92.8)	83 (95.4)	404 (91.2)	247 (91.1)	0.490	0.376	0.375	0.422	0.189	0.198
	< 2.5 kg	33 (7.2)	4 (4.6)	39 (8.8)	24 (8.9)						
**G6PD [a] deficiency phenotypes**	Normal	138 (56.6)	29 (58.0)	154 (55.2)	84 (53.8)	0.730	0.356	0.783	0.783	0.371	0.588
	Mildly deficient	84 (34.4)	18 (36.0)	89 (31.9)	55 (35.3)						
	Deficient	22 (9.0)	3 (6.0)	36 (12.9)	17 (10.9)						
**Sickle cell variants [c]**	Homozygous A	163 (72.4)	29 (69.0)	207 (80.9)	100 (71.9)	0.280	0.084	0.816	0.977	0.160	0.807
	Heterozygous AS/C	44 (19.6)	10 (23.8)	33 (12.9)	27 (19.4)						
	Homozygous S/C	18 (8.0)	3 (7.1)	16 (6.3)	12 (8.6)						
**Residence**	Rural	329 (71.7)	74 (85.1)	396 (89.2)	252 (92.0)	**<0.001**	**< 0.001**	**0.009**	**< 0.001**	0.269	0.058
	Urban	130 (28.3)	13 (14.9)	48 (10.8)	22 (8.0)						
**Socio-economic status**	Least poor	132 (28.8)	20 (23.0)	44 (9.9)	24 (8.8)	**< 0.001**	**< 0.001**	0.745	**< 0.001**	**0.003**	**< 0.001**
	Less poor	109 (23.7)	19 (21.8)	83 (18.7)	41 (15.0)						
	Poor	95 (20.7)	20 (23.0)	100 (22.5)	60 (21.9)						
	Poorer	76 (16.6)	17 (19.5)	105 (23.6)	62 (22.6)						
	Most poor	47 (10.2)	11 (12.6)	112 (25.2)	87 (31.8)						
**Malaria transmission season at birth**	High	258 (56.2)	43 (49.4)	220 (49.5)	148 (54)	0.210	**0.045**	0.243	0.563	0.983	0.455
	Low	201 (43.8)	44 (50.6)	224 (50.5)	126 (46)						
**Congenital abnormalities**	Yes	1 (0.2)	1 (1.1)	5 (1.1)	3 (1.1)	0.390	0.093	0.187	0.119	0.985	0.966
	No	458 (99.8)	86 (98.9)	439 (98.9)	271 (98.9)						
**Bed net use by infant [d]**	No/low usage	253 (55.1)	47 (54.0)	268 (60.4)	167 (61.0)	0.249	0.111	0.850	0.123	0.271	0.252
	High usage	206 (44.9)	40 (46.0)	176 (39.6)	107 (39.0)						

[a] G6PD = Glucose-6-phosphate dehydrogenase, [b] IQR = interquartile range, [c] Genotyping was carried out for first 900 infants enrolled into study, d No/low usage = infants who did not use or used bed nets less than 70% through the first year of life and High usage = infants who used bed nets 70% or more through the first year of life.
