# Supplementary material for: Determinants of the varied profiles of Plasmodium falciparum infections among infants living in Kintampo, Ghana
Source: Malar J. 2021 May 29;20:240. doi: 10.1186/s12936-021-03752-9 (PMC8164218; doi:10.1186/s12936-021-03752-9)
Supplement: Supplementary file 2 — Additional file 2: Table S2. Distribution of maternal and pregnancy factors between groups of infants. [file 12936_2021_3752_MOESM2_ESM.pdf]

Additional file 2. Distribution of maternal and pregnancy factors between groups of infants

**Characteristic**	**Category**	**Level**	**Parasite negative N = 459 n (%)**	**Only-asymptomatic N = 87 n (%)**	**Only-symptomatic N = 444 n (%)**	**Alternating N = 274 n (%)**	**Overall P-value**	**Parasite negative vs. Only-symptomatic P- value**	**Parasite negative vs. Only-asymptomatic P- value**	**Parasite negative vs. Alternating P-value**	**Only-asymptomatic vs. Only-symptomatic P- value**	**Only-asymptomatic vs. Alternating P- value**
**Age of mother**	**Years**	Median (IQR [e])	26 (22, 31)	27 (23, 32)	27 (21, 32)	26 (21, 31)	0.810	0.817	0.398	0.753	0.536	0.339
	**Age groups**	< 20	58 (12.7)	11 (12.6)	82 (18.8)	41 (15.2)	0.094	**0.017**	0.912	0.576	0.479	0.465
		20 – 29	272 (59.6)	52 (59.8)	227 (51.9)	156 (57.8)						
		30 – 40	116 (25.4)	21 (24.1)	110 (25.2)	70 (25.9)						
		> 40	10 (2.2)	3 (3.4)	18 (4.1)	3 (1.1)						
**Pregnancy**	**Gravidity**	Primagravidae	89 (19.4)	13 (14.9)	76 (17.1)	45 (16.4)	0.620	0.377	0.329	0.315	0.620	0.743
		Multigravidae	370 (80.6)	74 (85.1)	368 (82.9)	229 (83.6)						
	**Parity**	0	89 (19.4)	13 (14.9)	76 (17.1)	45 (16.4)	0.140	0.057	0.758	**0.032**	0.702	0.833
		1	85 (18.5)	17 (19.5)	76 (17.1)	44 (16.1)						
		2	93 (20.3)	15 (17.2)	73 (16.4)	38 (13.9)						
		3	62 (13.5)	14 (16.1)	52 (11.7)	47 (17.2)						
		≥ 4	130 (28.3)	28 (32.2)	167 (37.6)	100 (36.5)						
**Sickle cell trait/disease**		Yes	452 (99.6)	86 (98.9)	436 (99.5)	272 (100.0)	0.500	0.971	0.415	0.273	0.434	0.077
		No	2 (0.4)	1 (1.1)	2 (0.5)	0 (0.0)						
**ANC [a] attendance**	**Start (months)**	Median (IQR)	4 (3, 5)	4 (3, 5)	5 (3, 5)	4 (3, 6)	**<0.001**	**< 0.001**	0.072	**< 0.001**	0.595	0.192
	**Visits**	Median (IQR)	5 (4, 7)	4 (3, 5)	5 (3, 6)	4 (3, 6)	**<0.001**	**< 0.001**	**< 0.001**	**< 0.001**	**0.039**	0.618
**Tetanus immunization**	**Frequency**	Median (IQR)	1 (1, 2)	1 (0, 1)	1 (1, 2)	1 (0, 1)	**0.006**	0.146	**0.004**	**0.002**	0.089	0.407
**IPTp [b]**	**Doses**	0	28 (6.1)	6 (6.9)	16 (3.6)	17 (6.2)	**0.022**	**0.024**	0.177	**0.005**	0.495	0.870
		1	42 (9.2)	14 (16.1)	63 (14.2)	40 (14.7)						
		2	110 (24.0)	23 (26.4)	118 (26.6)	85 (31.1)						
		3	278 (60.7)	44 (50.6)	246 (55.5)	131 (48.0)						
	**DOTs [d]**	Yes	402 (93.3)	73 (90.1)	413 (95.8)	247 (95.7)	0.092	0.099	0.315	0.181	**0.032**	0.055
		No	29 (6.7)	8 (9.9)	18 (4.2)	11 (4.3)						
**ITN [c] use during pregnancy**	**Overall**	Yes	226 (49.5)	52 (61.2)	223 (50.6)	122 (45.4)	0.084	0.739	**0.047**	0.286	0.073	**0.011**
		No	231 (50.5)	33 (38.8)	218 (49.4)	147 (54.6)						
	**Rural**	Yes	152 (46.5)	43 (59.7)	191 (49.5)	108 (43.7)	0.110	0.594	**0.042**	0.511	0.079	**0.017**
		No	175 (53.5)	29 (40.3)	203 (51.5)	139 (56.3)						

	**Urban**	Yes	74 (56.9)	9 (69.2)	34 (68.1)	14 (63.6)	0.510	0.129	0.391	0.555	0.991	0.736
		No	56 (43.1)	4 (30.8)	15 (31.9)	8 (36.4)						
**Placental malaria**	**Overall**	Negative	299 (65.1)	58 (66.7)	271 (61.0)	165 (60.2)	**0.018**	0.136	0.097	**0.040**	**0.010**	**0.030**
		Past	138 (30.1)	23 (26.4)	143 (32.2)	84 (30.7)						
		Chronic	10 (2.2)	0 (0.0)	21 (4.7)	17 (6.2)						
		Acute	12 (2.6)	6 (6.9)	9 (2.0)	8 (2.9)						
	**Rural**	Negative	215 (65.3)	51 (68.9)	244 (61.6)	154 (61.1)	0.870	0.470	0.068	0.173	**0.026**	**0.040**
		Past	98 (29.8)	18 (24.3)	124 (31.3)	75 (29.8)						
		Chronic	9 (2.7)	0 (0.0)	19 (4.8)	16 (6.3)						
		Acute	7 (2.1)	5 (6.8)	9 (2.3)	7 (2.8)						
	**Urban**	Negative	84 (64.6)	7 (53.8)	27 (56.3)	11 (50.0)	0.530	0.141	0.812	0.347	0.236	0.857
		Past	40 (30.8)	5 (38.5)	19 (39.6)	9 (40.9)						
		Chronic	1 (0.8)	0 (0.0)	2 (4.2)	1 (4.5)						
		Acute	5 (3.8)	1 (7.7)	0 (0.0)	1 (4.5)						
**Delivery**	**Gestational age**	Pre-term	6 (1.3)	1 (1.1)	3 (0.7)	0 (0.0)	0.570	0.572	0.804	0.164	0.608	0.161
		Term	399 (87.1)	78 (89.7)	385 (86.7)	238 (87.8)						
		Late	53 (11.6)	8 (9.2)	56 (12.6)	33 (12.2)						
	**Place**	Health facility	320 (69.7)	59 (67.8)	259 (58.3)	149 (54.4)	**0.002**	**0.004**	0.806	**< 0.001**	0.268	0.081
		Private maternity home	17 (3.7)	2 (2.3)	28 (6.3)	12 (4.4)						
		Home/TBA [f]	121 (26.4)	26 (29.9)	156 (35.1)	113 (41.2)						
		On the way to HF/PMH/TBA [f]	1 (0.2)	0 (0.0)	1 (0.2)	0 (0.0)						

[a] ANC = antenatal care, [b] IPTp = intermittent preventive treatment during pregnancy, [c] ITN = insecticide treated bed-net, [d] DOTs = direct observed therapy, [e] IQR = interquartile range, [f] HF = health facility PMH = private maternity home, TBA= traditional birth attendant
