# Supplementary material for: Determinants of the varied profiles of Plasmodium falciparum infections among infants living in Kintampo, Ghana
Source: Malar J. 2021 May 29;20:240. doi: 10.1186/s12936-021-03752-9 (PMC8164218; doi:10.1186/s12936-021-03752-9)
Supplement: Supplementary file 3 — Additional file 3: Table S3. Parasite negative versus only-asymptomatic*, only-symptomatic* and alternating*. [file 12936_2021_3752_MOESM3_ESM.pdf]

Additional file 3. Parasite negative versus only-asymptomatic*, only-symptomatic* and alternating*

Characteristic	Level	Parasite negative group N = 459 n (%)	Parasite positive group N= 805 n (%)	p-value
**Residence**	Rural	329 (71.7)	722 (89.7)	**< 0.001**
	Urban	130 (28.3)	83 (10.3)	
**Socio-economic status**	Least poor	132 (28.8)	88 (10.9)	**< 0.001**
	Less poor	109 (23.7)	143 (17.8)	
	Poor	95 (20.7)	180 (22.4)	
	Poorer	76 (16.6)	184 (22.9)	
	Most poor	47 (10.2)	210 (26.1)	
**Parity**	0	89 (19.4)	134 (16.6)	**0.024**
	1	85 (18.5)	137 (17.0)	
	2	93 (20.3)	126 (15.7)	
	3	62 (13.5)	113 (14.0)	
	≥ 4	130 (28.3)	295 (36.6)	
**IPTp [a] doses**	0	28 (6.1)	39 (4.9)	**0.004**
	1	42 (9.2)	117 (14.6)	
	2	110 (24.0)	226 (28.1)	
	3	278 (60.7)	421 (52.4)	
**Delivery place**	Health facility	320 (69.7)	467 (58.0)	**0.001**
	Private maternity home	17 (3.7)	42 (5.2)	
	Home/TBA [b]	121 (26.4)	295 (36.6)	
	On the way to HF/PMH/TBA [c]	1 (0.2)	1 (0.1)	

* = Parasite positive group. [a] IPTp = intermittent preventive treatment during pregnancy, [b] TBA= traditional birth attendant, [c] HF = health facility, PMH = private maternity home.
