# Supplementary material for: Determinants of the varied profiles of Plasmodium falciparum infections among infants living in Kintampo, Ghana
Source: Malar J. 2021 May 29;20:240. doi: 10.1186/s12936-021-03752-9 (PMC8164218; doi:10.1186/s12936-021-03752-9)

# Additional file 4: Sickle cell variation and time to first malaria infection

### A. Only-asymptomatic

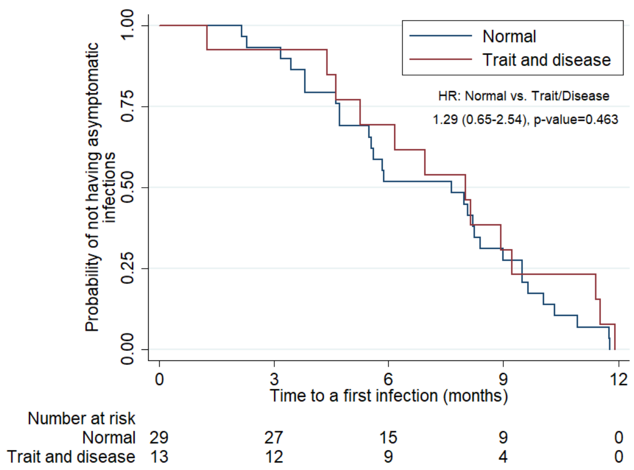


### B. Only-symptomatic

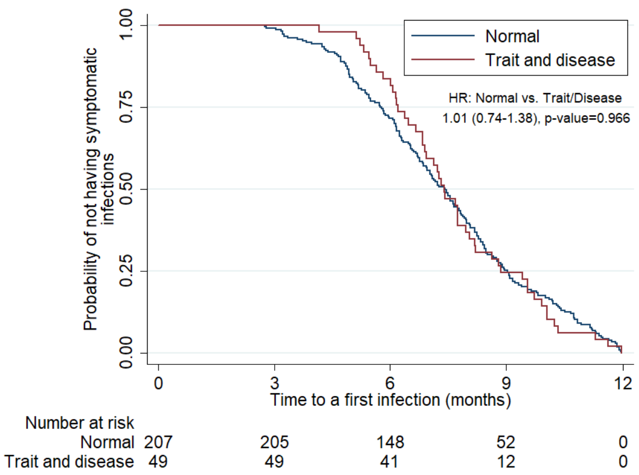


### C. Alternating

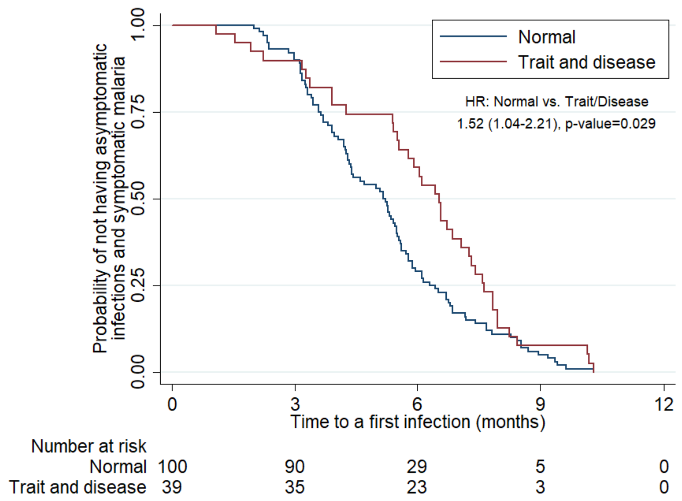

Supplement: Supplementary file 4 — Additional file 4: Figure S1. Sickle cell variation and time to first malaria infection. [file 12936_2021_3752_MOESM4_ESM.pdf]
