# Supplementary material for: Determinants of the varied profiles of Plasmodium falciparum infections among infants living in Kintampo, Ghana
Source: Malar J. 2021 May 29;20:240. doi: 10.1186/s12936-021-03752-9 (PMC8164218; doi:10.1186/s12936-021-03752-9)

Additional file 5: G6PD deficiency and time to first malaria infection

A. Only-asymptomatic

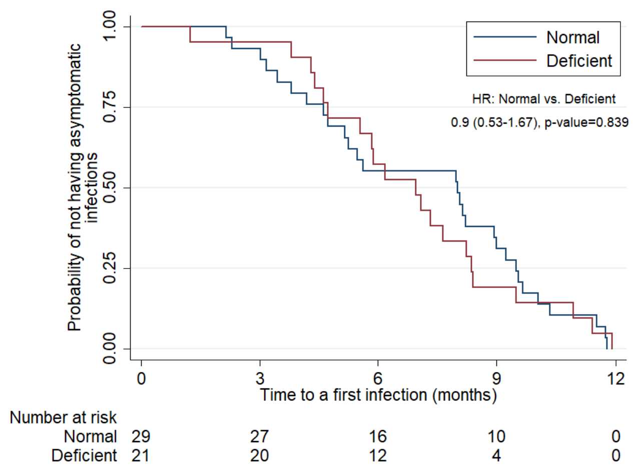

B. Only-symptomatic

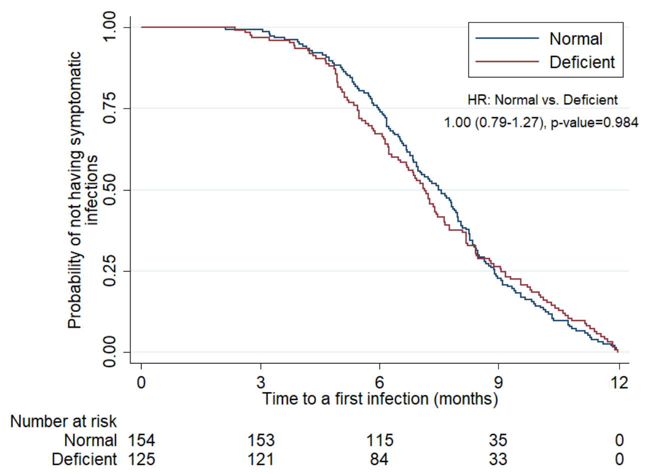

C. Alternating

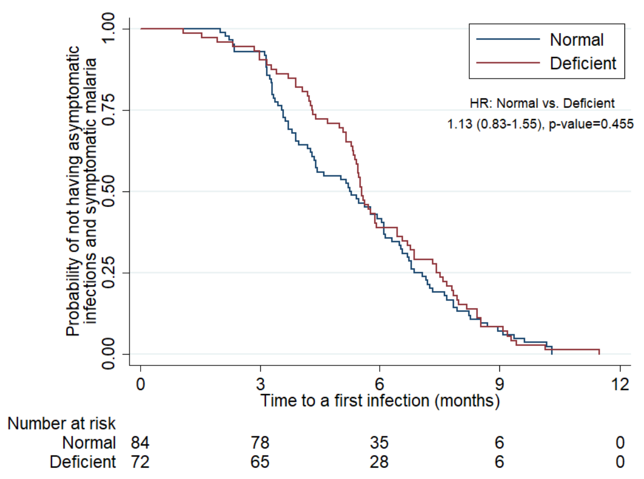

Supplement: Supplementary file 5 — Additional file 5: Figure S2. G6PD deficiency and time to first malaria infection. [file 12936_2021_3752_MOESM5_ESM.pdf]
